# Disturbed left ventricular inflow and ejection pattern in corrected atrioventricular septal defect patients assessed by 4DFlow MRI and particle tracing

**DOI:** 10.1186/1532-429X-17-S1-P59

**Published:** 2015-02-03

**Authors:** Emmeline Calkoen, Patrick J de Koning, Rob J van der Geest, Albert de Roos, Arno Roest, Jos J Westenberg

**Affiliations:** 1 Pediatric Cardiology, Leiden University Medical Center, Leiden, Netherlands; 2 Radiology, Leiden University Medical Center, Leiden, Netherlands

## Background

The normal pattern of left ventricular (LV) inflow and ejection of affects the efficiency of cardiac pumping performance. Altered inflow direction due to a corrected atrioventricular septal defect (AVSD) may disturb this pattern leading to decreased efficiency. We aimed to quantitatively describe the LV blood flow pattern using 4-dimensional velocity-encoded cardiac magnetic resonance imaging (4DFlow MRI) and particle tracing in healthy volunteers and corrected AVSD patients.

## Methods

32 patients (age 25±14 years) and 30 healthy volunteers (age 26±12 years) were included. Whole-heart 4D Flow MRI was performed on 3Tesla MRI with free breathing, three-directional velocity encoding of 150cm/s in all directions, spatial resolution 2.3×2.3×3.0-4.2mm^3^ and 30 phases reconstructed over one cardiac cycle. At end-diastole the LV was evenly filled with particles and subsequently tracked by backward and forward particle tracing to analyze the LV 4-componental flow as introduced by Eriksson [JCMR, 2010] discriminating 1. direct flow entering and leaving the LV within one cycle, 2. retained flow entering during diastole but remaining in LV during next systole, 3. delayed ejected flow already in LV before diastole and leaves LV during systole 4. residual volume. Regurgitant flow in patients was added as a fifth component. The path of inflowing particles (i.e. direct and retained flow) during diastole was evaluated using the AHA 16-segment LV cavity model. Difference in componental percentage and in particle percentage (pp; amount of particles per segment as percentage of the sum of particles) between patient and controls were compared using unpaired t-tests.

## Results

**Figure 1:**
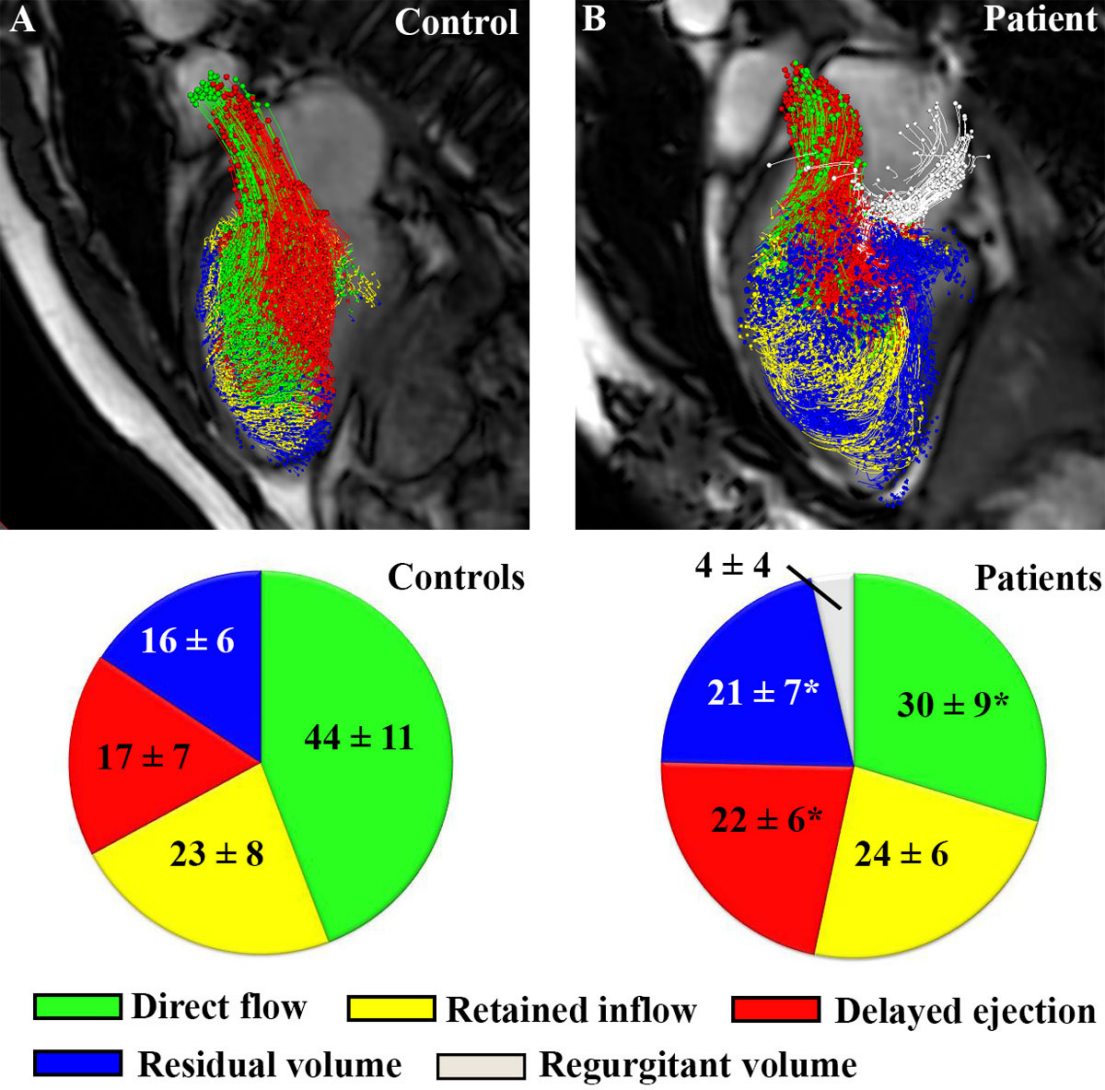
Example of a control and patient at an early systolic phase. Pie chart presents component percentage of left ventricular flow, with * indicating P<0.01. Direct flow enters and leavs the LV within one cycle. Retained flow enters during diastole but remains in LV during next systole. Delayed ejected flow is already in LV before diastole and leaves LV during systole. Residual volume remains in LV during whole cardiac cycle. Regurgitant volume contains blood entering the left atrium during systole.

**Figure 2:**
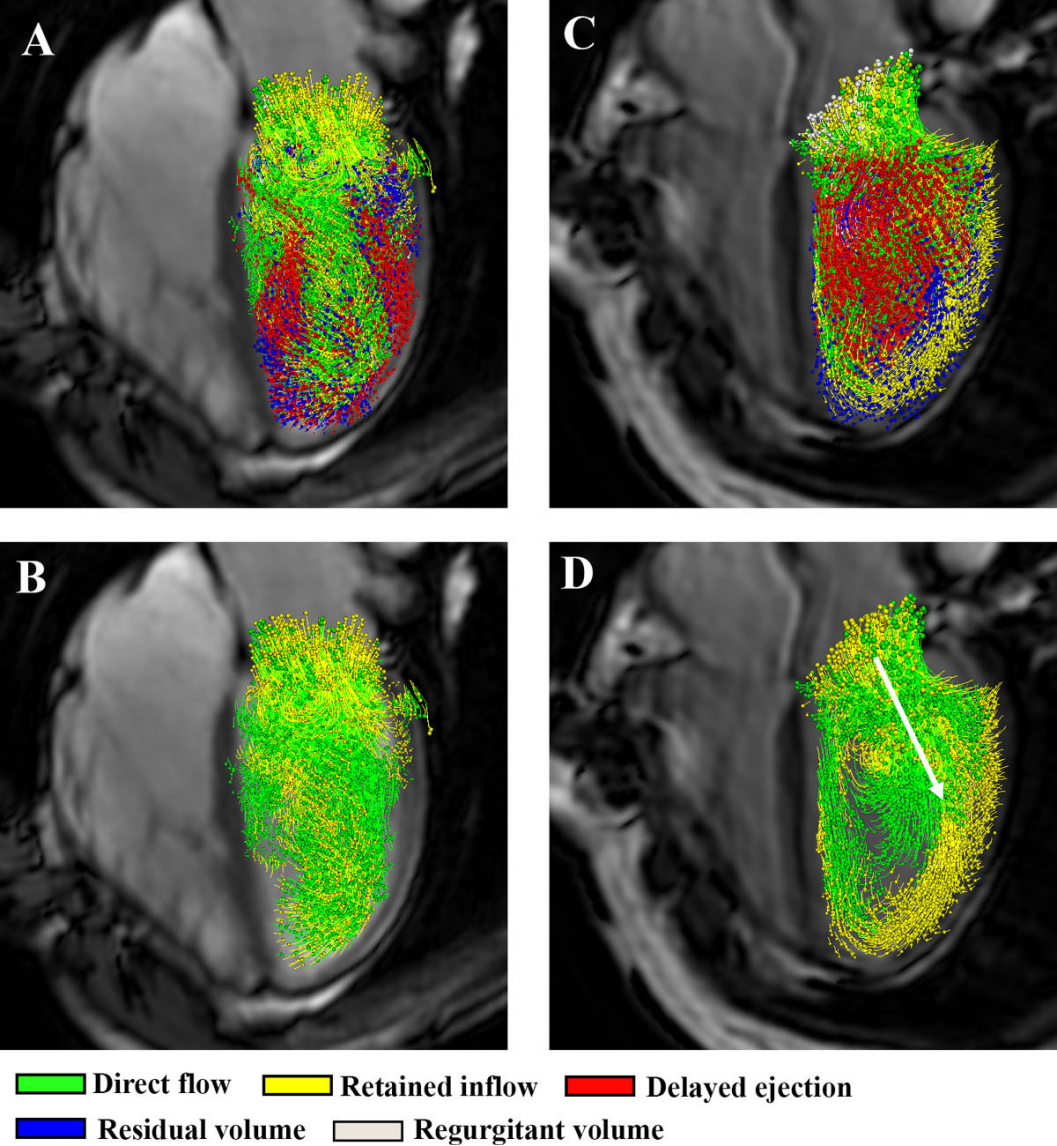
Particle tracing during diastole in a control (A and B) and a patient (C and D), with in B and D more lateral and apical flow contribution of particles belonging to the retained flow.

Patients showed a smaller percentage of direct flow compared to volunteers (30±9% versus 44±11%, p<0.001) and larger percentage of delayed ejected flow (22±6% versus 17±7%, p=0.004) and residual volume (21±7% versus 16±6%, p=0.002)(Figure 1). In the LV apex a similar pp of direct flow (4±4% versus 4±4%, p=0.96), but significantly higher retained flow (7±4% versus 5±2%, p=0.009) was observed. In the mid-lateral segments, decreased pp of direct flow (13±5% versus 16±5%, p=0.015) and increased retained flow (11±4% versus 7±4%, p<0.001) was observed (Figure 2).

## Conclusions

Particle tracing and 4DFlow MRI enable quantitative assessment of altered LV filling and ejection patterns after AVSD correction, with less direct flow and more residual volume in patients versus volunteers, and an increase in (retained) flow directed to the mid-lateral and apical segments, which may contribute to a decreased cardiac pumping efficiency.

## Funding

E.E. Calkoen is financially supported by a grant from the Willem-Alexander Kinder- en Jeugdfonds, J.J.M. Westenberg is financially supported by a grant from the Dutch Technology Foundation (STW), project number 11626.

